# Negative Pressure Wound Therapy with Instillation as Conservative Management for a Large Abdominal Wound Following Incarcerated Parastomal Hernia Repair

**DOI:** 10.7759/cureus.3728

**Published:** 2018-12-13

**Authors:** Christina Raykha, Amir Sameh Riad Botros, Yasmine Roden, Peter Murchan

**Affiliations:** 1 General Surgery, South Tipperary General Hospital, Clonmel, IRL

**Keywords:** parastomal hernia, mesh infection, hernia repair, negative pressure wound therapy, vac therapy, npwt, secondary intention healing

## Abstract

Parastomal herniation is a common complication following stoma creation, necessitating surgical repair in up to one-quarter of cases, including emergency cases of incarceration or strangulation. Following hernia repair with or without mesh placement, surgical sites are at risk of infection post-operatively and this is typically resolved by removing the mesh, which can be technically challenging. Few studies have assessed conservative management options for these types of cases. Here, we present a case where negative pressure wound therapy (NPWT) with instillation was utilized for secondary intention healing of a large abdominal wound (20 cm x 23 cm x 5 cm) following mesh infection post-parastomal hernia repair. The patient’s wound was completely healed after eight weeks and she had no long-term complications at the one-year follow-up. NPWT with instillation is an option for the conservative management of large abdominal wounds, which can be considered on a case-by-case basis.

## Introduction

Parastomal herniation is one of the most common complications of an end colostomy, with a prevalence ranging from 5% - 50% [1]. Approximately 25% of parastomal hernia cases require surgical repair, however, this procedure is associated with a high recurrence rate [2-3] and for this reason, conservative management is often recommended to patients. Conservative management involves patient education, weight loss, and possibly a hernia belt to prevent further expansion of the hernia itself. If the parastomal hernia becomes symptomatic for the patient, surgery is considered, and in emergent cases, such as incarceration or strangulation, surgical repair is required. The gold standard currently is laparoscopic repair with a prosthetic mesh, which has been shown to reduce parastomal hernia recurrence rates relative to suture repair [2-3]. Unfortunately, this preferred repair option is not without complications, which includes pain, mesh migration, adhesions, mesh rejection, fistula formation, and surgical site infections [2,4].

Surgical site infections are some of the most common surgical complications, with an incidence of approximately 12% - 15% in abdominal surgeries [1,5]. Following hernia repair with mesh, mesh infection is reported to occur in 2.3% of the cases [3]. Traditionally, mesh infection led to mesh removal rather than conservative management [6], however, Meagher et al. report successful conservative management of hernia repair and mesh infection using percutaneous drainage and antibiotic irrigation [7]. Other studies have assessed negative pressure wound therapy (NPWT), which has revolutionized wound healing, especially post-operatively in recent decades, as an adjunct to primary closure in hernia repair [8-10]. NPWT use has been associated with reduced hernia recurrence rates and surgical site infections as well as improved mesh salvage rates and post-repair complication rates [8,10].

The success of NPWT is attributed to its ability to increase granulation tissue and angiogenesis while decreasing edema and bacterial colonization [11]. One study found that NPWT effectively reduced wound size by approximately 4% per day [12], and other studies have found a much lower incidence of complications with the use of NPWT relative to traditional dressings [10,13]. It is of no surprise that NPWT has been becoming more popular postoperatively in abdominal surgeries. More recently, studies are making use of NPWT with instillation and dwell, which allows a sterile solution to irrigate and clean the wound before applying negative pressure (vacuum) to remove the debris and exudate [14].

Here, we present a case of a patient with an incarcerated parastomal hernia, which required an emergency laparotomy with mesh placement. The mesh became infected, which led to the re-exploration of the wound and the use of the NPWT with instillation system to allow full healing by secondary intention. Approximately two months later, the wound had healed completely.

## Case presentation

The patient is a 61-year-old lady who presented to the emergency department (ED) with a one-day history of severe lower abdominal pain and nausea. She had previously undergone pelvic exenteration with a permanent end colostomy for stage IIIb cervical cancer. Approximately two years later, she developed a parastomal hernia and had been managing it conservatively. On this admission, she was diagnosed with an incarcerated parastomal hernia.

Emergency laparotomy was performed with the resection of more than one-and-a-half meters of gangrenous bowel and the stoma was re-fashioned more superiorly to its original location. An UltraPro (Ethicon, Inc Somerville, NJ, US) surgical mesh was applied with primary closure. The surgery was completed successfully and the patient was then transferred to the ICU for further monitoring.

Approximately one week later, the patient was pyrexial and had not responded to treatment sufficiently. On CT imaging, there was a five-centimeter collection identified over the mesh. The wound was then opened, re-explored in the ICU, and debridement was completed. Initially, the wound dimensions were 20 cm x 23 cm x 5 cm, with exposure down to the rectus sheath. To ensure healing, the VeraFlo™ Vacuum (KCI Technology, San Antonio, TX, US) with instillation dressing was commenced at -125 mmHg with a sterile saline solution being cycled for three hours prior to vacuum application. The dressing was changed three times weekly and prior to replacement, the dressing was soaked in saline solution for 20 minutes. After seven weeks, the wound had reduced to 9 cm x 5 cm x 1.6 cm. There was also a dramatic decrease in the amount of exudate and debris over the seven weeks, which resulted in a decreased instillation volume. During this time, the patient also reported improved pain control relative to the post-operative period.

**Figure 1:**
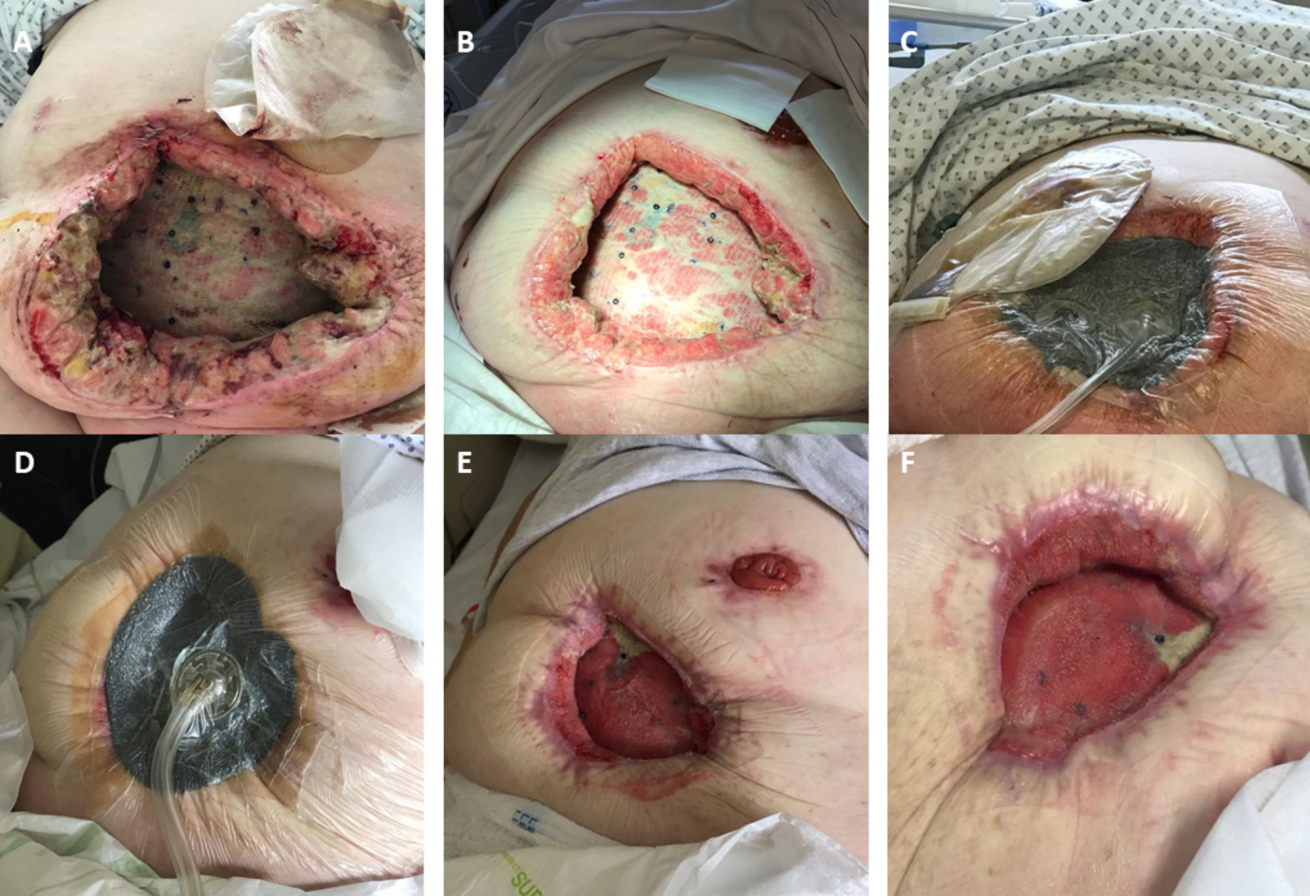
Progression of wound following debridement with VeraFlo dressing A) Day 1 pre-VeraFlo dressing. Wound after opening in ICU B) Wound after Day 4 on negative pressure wound therapy (NPWT) with instillation (VeraFlo). C) VeraFlo dressing in situ on Day 4 D) VeraFlo dressing in situ on Day 7 E) Wound on Day 31 F) Wound on Day 49, prior to switching to ActiVAC therapy VeraFlo™: KCI Technology, San Antonio, TX, US

The dressing was then switched to the ActiVAC™ therapy (KCI Technology, San Antonio, TX, US), a more compact, portable version of NPWT, which allowed the patient more freedom to mobilize. This helped to facilitate the discharge planning process. Within the following two weeks, the wound had completely healed (Figure 1) without the necessity of a skin graft or tertiary closure. One year later, the patient had not experienced any late complications on outpatient follow-up.

The authors would like to disclose that KCI Technology provided financial support for ASRB to attend and present at a conference previously.

## Discussion

To our knowledge, this is the first clinical case study utilizing the NPWT with instillation system for the complete healing of a large abdominal wall defect following an incarcerated parastomal hernia repair with mesh infection. The removal of gangrenous bowel and the emergency setting for this case were risk factors for postoperative complications [9], which have been previously published following hernia repair or abdominal wall surgery. These risk factors include age > 70 years, concurrent bowel surgery, previous hernia repair, diameter > 10 cm, emergency surgery, and previous cancer diagnosis [9,15]. After parastomal hernia repair, more than 60% were shown to have at least one postoperative complication.

Secondary intention healing application

There are few studies where NPWT was used or assessed for complete secondary intention healing of surgical wounds [16-19]. A Cochrane review was published on the use of NPWT for the healing of wounds by secondary intention; however, open abdominal wounds were excluded from this study [16]. Unfortunately, no conclusive results were made from this Cochrane review, which highlights the need for prospective, large, high-quality trials to assess NPWT therapy relative to conventional dressings. One study found that 0.4 per 1000 surgical wounds were healing by secondary intention in the UK and, of these, NPWT was being utilized in 6% of the cases [19]. NPWT has also shown its efficacy in cases of severe abdominal sepsis with full wound closure occurring after eight weeks in a patient post-Caesarean section [18] and in cases of tertiary closure post-surgically with abdominal sepsis [13]. Another study assessed the combination of noncontact, low-frequency ultrasound with NPWT (no instillation) and found that wound healing was markedly improved with combination therapy [17].

NPWT with instillation applications

While NPWT has certainly shown clinical efficacy over time, NPWT with instillation, which delivers a sterile solution to the wound to aid in irrigation and clearance of debris, has also been used successfully in various applications, including abdominal surgeries [14]. This technology was utilized for wound dehiscence following abdominal wall surgery and was shown to decrease the number of subsequent surgeries as well as the length of treatment [9]. Another study in Mexico assessed NPWT with instillation in abdominal wall reconstruction cases relative to conventional treatment and found that there was an increased rate of fascial closure, shorter intensive care unit (ICU) stay, and decreased mortality [13]. Additionally, in this study and others, it was found that there were no complications in the NPWT with instillation cases [13]. When compared to NPWT alone (without instillation), Omar et al. reported that patients receiving NPWT with instillation had a shorter hospital stay and faster wound healing [20]. Despite this being a smaller scale study with 10 patients in each group, these findings further highlight the benefits of the NPWT with instillation system, even relative to NPWT alone [20].

## Conclusions

This case report demonstrates the successful use of the NPWT with instillation system to aid in complete secondary intention healing of a large abdominal wall defect following parastomal hernia repair. The NPWT with instillation system has been shown in numerous studies to decrease hospital stay and prevent complications, thereby positively affecting patient outcomes, including pain control. There is a need for larger, randomized trials to assess the effectiveness of NPWT relative to NPWT with instillation in terms of patient satisfaction, pain control, wound healing progress, complication rates, and hospital costs in various clinical applications. Nonetheless, recent literature, in addition to this case report, has successfully highlighted the benefits of the NPWT with instillation system, facilitating complete healing of the large abdominal wound post a mesh infection.
